# Clinical evaluation of noninvasive prenatal testing for sex chromosome aneuploidies in 9,176 Korean pregnant women: a single-center retrospective study

**DOI:** 10.1186/s12884-024-06275-8

**Published:** 2024-01-31

**Authors:** Hyunjin Kim, Ji Eun Park, Kyung Min Kang, Hee Yeon Jang, Minyeon Go, So Hyun Yang, Jong Chul Kim, Seo Young Lim, Dong Hyun Cha, Jungah Choi, Sung Han Shim

**Affiliations:** 1 Center for Genome Diagnostics, CHA Biotech Inc, Seoul, 06125 Republic of Korea; 2 Department of Obstetrics and Gynecology, CHA Gangnam Medical Center, CHA University, Seoul, 06135 Republic of Korea; 3 College of Liberal Art, CHA University, Pocheon, Gyeonggi, Republic of Korea; 4 Department of Biomedical Science, College of Life Science, CHA University, Seongnam, 13488 Republic of Korea

**Keywords:** Noninvasive prenatal testing, Sex chromosome aneuploidies, Positive predictive value, Prenatal diagnosis, Genetic counseling

## Abstract

**Background:**

To evaluate the clinical significance of noninvasive prenatal testing (NIPT) for detecting fetal sex chromosome aneuploidies (SCAs) in Korean pregnant women.

**Methods:**

We retrospectively analyzed NIPT data from 9,176 women with singleton pregnancies referred to the CHA Biotech genome diagnostics center. Cell-free fetal DNA (cffDNA) was extracted from maternal peripheral blood, and high-throughput massively parallel sequencing was conducted. Subsequently, the positive NIPT results for SCA were validated via karyotype and chromosomal microarray analyses.

**Results:**

Overall, 46 cases were SCA positive after NIPT, including 20, 12, 8, and 6 for Turner, triple X, Klinefelter, and Jacob syndromes, respectively. Among 37 women with invasive prenatal diagnosis, 19 had true positive NIPT results. The overall positive predictive value (PPV) of NIPT for detecting SCAs was 51.35%. The PPV was 18.75% for Turner, 88.89% for triple X, 71.43% for Klinefelter, and 60.00% for Jacob’s syndromes. NIPT accuracy for detecting sex chromosome trisomies was higher than that for sex chromosome monosomy (*P* = 0.002). No significant correlation was observed between fetal SCA incidence and maternal age (*P* = 0.914), except for the borderline significance of Jacob’s syndrome (*P* = 0.048). No significant differences were observed when comparing NIPT and karyotyping validation for fetal SCA according to pregnancy characteristics.

**Conclusion:**

Our data suggest that NIPT can reliably screen for SCAs, and it performed better in predicting sex chromosome trisomies compared with monosomy X. No correlation was observed between maternal age and fetal SCA incidence, and no association was observed between different pregnancy characteristics. The accuracy of these findings requires improvements; however, our study provides an important reference for clinical genetic counseling and further management. Larger scale studies, considering confounding factors, are required for accurate evaluation.

**Supplementary Information:**

The online version contains supplementary material available at 10.1186/s12884-024-06275-8.

## Background

Sex chromosome aneuploidies (SCAs) result from an abnormal number of X and/or Y-chromosomes beyond the typical female (XX) or male (XY) configurations, such as Turner (45,X), triple X (47,XXX), Klinefelter (47,XXY), and Jacob’s (47,XYY) syndromes [1]. Approximately one in every 500 newborns has an SCA condition, surpassing the prevalence of common trisomy 21. Clinical symptoms of SCA vary depending on the type of sex chromosome mutation, ranging from mild or asymptomatic to severe functional impairment, such as developmental delays, infertility, behavioral problems, or learning disabilities [2, 3]. As effective treatments are lacking, early intervention, including hormonal therapy through screening and prenatal detection of SCA, is important [3, 4].

An effective and safe method for detecting fetal chromosomal abnormalities is required because of the increasing number of pregnant women at advanced maternal age (AMA) resulting from socioeconomic factors. Invasive prenatal tests offer accurate diagnosis; however, invasive procedures may cause fetal loss, infections, and maternal anxiety [4, 5]. Since the discovery of cell-free fetal DNA (cffDNA) by Lo et al*.* and the introduction of massively parallel sequencing, noninvasive prenatal testing (NIPT) has become a first-tier screening method for fetal chromosomal aneuploidies [6, 7]. Numerous NIPT studies have revealed that compared with biochemical screening methods, NIPT exhibits high sensitivity and specificity in detecting trisomies 21, 18, and 13, with low false positive and false negative rates [8–10]. In a meta-analysis conducted by Phillips et al., NIPT exhibited a sensitivity of 99.3%, 97.4%, and 97.4%, and specificity of 99.9%, 99.9%, and > 99.9% for trisomies 21, 18, and 13, respectively [11]. Recently, NIPT has expanded to include SCAs, rare chromosomal aneuploidies, and some specific microdeletion syndromes [12–14]. However, its clinical application faces challenges because the PPV of SCA varies depending on the study groups, ranging from 26.8 to over 90% [1, 5, 12–24]. Additionally, compared with reports on NIPT performance for common trisomy, those on rare chromosomal aneuploidies and selected microdeletion syndromes, based on actual clinical data, are limited, resulting in less information on their detection accuracy [12–14, 25]. This leads to challenges in clinical genetic counseling, resulting in maternal anxiety and unnecessary invasive diagnostic procedures; therefore, further studies on NIPT validation are needed. In this study, we aimed to retrospectively evaluate the clinical significance of NIPT for SCAs in Korean pregnant women.

## Materials and methods

### Patients

This retrospective study aimed to evaluate the clinical significance of NIPT for SCAs in Korean pregnant women. We retrospectively collected data from pregnant women who underwent NIPT at the genome diagnostics center for CHA Biotech Inc. in Seoul, Republic of Korea, between July 2018 and December 2022. The exclusion criteria included pregnant women with multiple pregnancies or vanishing twins, fetal chromosomal abnormalities other than SCAs, and no reported NIPT results. The Institutional Review Board of CHA Gangnam Medical Center, CHA University, approved the study (GCI 2023–03-006).

### NIPT

Maternal peripheral blood (10 mL) was collected in Cell-Free DNA BCT™ tubes (Streck, Omaha, NE, USA). The blood samples were centrifuged at 1200 × g for 10 min at 4 °C, followed by a secondary centrifugation of the collected plasma portion at 16,000 × g for 10 min at 4 °C. The cell-free DNA (cfDNA) was extracted from 1 mL of plasma using the QIAamp Circulating Nucleic Acid kit (Qiagen, Hilden, Germany). DNA libraries were constructed using the Ion Plus Fragment Library kit (Thermo Fisher, Waltham, CA, USA) following the manufacturer’s instructions. Subsequently, DNA libraries were analyzed using the Ion S5™ XL System (Thermo Fisher, Waltham, CA, USA) with an average 0.3X sequencing coverage depth. A total of 14 cfDNA samples were loaded onto an Ion 540™ Chip Kit (Thermo Fisher, Waltham, CA, USA). The raw reads of each sample were above 5 million, and the rates of uniquely mapped reads were above 65.0%. The Z-score for each chromosome was calculated by referencing the normalized chromosome representation. Chromosomes with absolute Z-scores > 3 were identified as having chromosome aneuploidies. The absence of results in a sample was attributed to insufficient (< 4.0%) cffDNA fraction, unusually high variation in cffDNA counts, or failure to pass the quality control measures.

### Invasive prenatal diagnosis

Pregnant women with NIPT-positive results for SCA underwent genetic counseling and were informed about undergoing invasive prenatal diagnosis. Chromosome karyotype analysis was performed using cells obtained from the amniotic fluid according to standard protocols. The amniotic fluid was centrifuged, the precipitated cells were collected, and the cells were cultured in a BIO-AMFTM medium (Biological Industries, USA). After G-banding, each slide was observed under a microscope, and 20 to 30 cells were counted and analyzed. CMA analysis was performed using CytoScan 750 K (Affymetrix, Santa Clara, CA, USA) according to the manufacturer’s protocol. The array is characterized with > 750,436 CNV markers, including 200,436 SNP probes and > 550,000 non-polymorphism probes. Data were visualized and analyzed with the Chromosome Analysis Suite software package (Affymetrix) using Human Genome build hg19.

### Statistical analysis

All statistical analyses were conducted using R v.4.1.1 (https://www.r-project.org/) software. The chi-square test was used to assess the association between sex chromosome trisomy and monosomy X, and compare the frequencies of SCA among pregnant women in different age groups. Fisher’s exact test was used when the frequency count was < 5. Statistical significance was set at *P* < 0.05.

## Results

### Patient characteristics

We enrolled 9,176 women with singleton pregnancies between July 2018 and December 2022. The mean maternal age at the time of NIPT was 36.29 years, with a mean gestational age of 12^+3^ weeks and mean body mass index (BMI) of 22.21. The majority (86.24%) of pregnant women underwent NIPT between 9 and 12^+6^ weeks, and 58.49% had BMIs of 18.5–22.9. Among the participants, 6,571 (71.61%) had AMA (age ≥ 35 years), and 2,403 (26.19%) had in vitro fertilization (IVF) pregnancies (Table 1).

**Table 1:** Patient characteristics of 9,176 singleton pregnancies undergoing NIPT for chromosomal aneuploidy

Characteristics	Number (%)
**Total**	9,176 (100.00)
**Singleton pregnancy**	9,176 (100.00)
**Maternal age (years)**	
Mean (± SD)	36.29 (± 3.63)
Min–max	20–48
< 35	2,605 (28.39)
≥ 35	6,571 (71.61)
**Gestational age at NIPT (weeks)**	
Mean (± SD)	12^+3^(± 1^+3^)
Min–max	9^+0^–31^+0^
9–12^+6^	7,913 (86.24)
13–14^+6^	634 (6.90)
15–19^+6^	601 (6.55)
≥ 20	28 (0.31)
**BMI**	
Mean (± SD)	22.21(± 3.28)
Min–max	14.18–41.91
< 18.5	738 (8.04)
18.5–22.9	5367 (58.49)
23.0–24.9	1463 (15.94)
25.0–29.9	1317(14.35)
≥ 30	291 (3.17)
**Method of conception**	
Natural	6,773 (73.81)
IVF	2,403 (26.19)

*SD* Standard deviation, *BMI* Body mass index, *IVF* In vitro fertilization, *NIPT* noninvasive prenatal testing

### Detective efficiency of SCAs using NIPT

Among 9,176 cases, 46 (0.50%) had SCA-positive NIPT results, including 20 cases of 45,X, 12 of 47,XXX, 8 of 47,XXY, and 6 of 47,XYY. Among the pregnant women, 37 (80.43%) accepted the invasive diagnosis, and 9 refused further prenatal diagnosis or were lost to follow-up (Table 2).

**Table 2:** A comparison of the positive NIPT results for SCAs compared with karyotyping in pregnant women

SCA type	NIPT positive (N)	Prenatal diagnostic validated							Incidence (%)
		**TP (N)**	**FP (N)**	**Unverified (N)**	**Sensitivity (%)**	**Specificity (%)**	**PPV (%)**	**FPR (%)**	
45,X	20	3	13	4	100.00	99.86	18.75	0.14	0.033
47,XXX	12	8	1	3	100.00	99.99	88.89	0.01	0.087
47,XXY	8	5	2	1	100.00	99.98	71.43	0.02	0.054
47,XYY	6	3	2	1	100.00	99.98	60.00	0.02	0.033
Overall	46	19	18	9	100.00	99.80	51.35	0.20	0.207

*SCA* sex chromosome aneuploidy, *TP* true positive, *FP* false positive, *PPV* positive predictive value, *FPR* false positive rate, *NIPT* noninvasive prenatal testing

After prenatal diagnosis, 19 and 18 women had true and false positive NIPT results, respectively. The sensitivity of NIPT for 45,X, 47,XXX, 47,XXY, and 47,XYY were all 100%. The specificity for 45,X, 47,XXX, 47,XXY, and 47,XYY, were 99.86%, 99.99%, 99.98%, and 99.98%, respectively. The overall PPV of NIPT for detecting fetal SCAs was 51.35% (19/37). The PPVs for 45,X, 47,XXX, 47,XXY, and 47,XYY, were 18.75% (3/16), 88.89% (8/9), 71.43% (5/7), and 60.00% (3/5), respectively. The false positive rates for 45,X, 47,XXX, 47,XXY, and 47,XYY, were 0.14%, 0.01%, 0.02%, and 0.02%, respectively. We evaluated the associations between sex chromosome trisomy and monosomy X. Sex chromosome trisomy had significantly higher detection rates (16/21) than sex chromosome monosomy (3/16) (χ2 = 9.805, *P* = 0.002, odds ratio (OR) = 12.65, 95% confidence interval (CI) -2.28 to 99.02).

### Comparative evaluation of fetal SCA incidence by maternal age

We divided the participants into four groups based on their age (≤ 29, 30–34, 35–39, and ≥ 40 years) to compare fetal SCA incidence by maternal age. The PPV increased with maternal age groups and was the highest among the ≥ 40-year-old group (Table 3). However, no significant difference was observed between different maternal age groups and fetal SCA incidence (*P* = 0.914), but there were mild differences between 47,XYY incidence and maternal age groups (*P* = 0.048).

**Table 3:** Comparison of the incidence of fetal SCAs among different age groups

Age groups	No.of patients	45,X^a^	47,XXX^b^	47,XXY^c^	47,XYY^d^	All SCA^e^	PPV(%)
≤ 29	352	0	0	0	0	0	0
30–34	2,253	0	3	0	3	6	46.15
35–39	4,841	3	3	4	0	10	55.56
≥ 40	1,730	0	2	1	0	3	75.00
Total	9,176	3	8	5	3	19	51.35

*PPV* positive predictive value, *SCA* sex chromosome aneuploidy

^a^*P* = 0.637 for incidence of 45,X

^b^*P* = 0.676 for incidence of 47,XXX

^c^*P* = 0.585 for incidence of 47,XXY

^d^*P* = 0.048 for incidence of 47,XYY

^e^*P* = 0.914 for incidence of All SCA

### Comparative evaluation of the NIPT and prenatal diagnosis for fetal SCAs based on pregnancy characteristics

We also compared SCA-positive NIPT results with invasive prenatal diagnoses based on different pregnancy characteristics, including maternal age, gestational age, maternal BMI, conception method, and clinical indications to determine the correlation between pregnancy characteristics and fetal SCA incidence (Table 4). No significant difference was associated with maternal age (*P* = 0.142), gestational age (*P* = 0.929), maternal BMI (*P* = 0.531), conception method (*P* = 0.672), and clinical indications (*P* = 0.147).

**Table 4:** Comparison between different stratified pregnancy characteristics with the results of prenatal diagnostic validation

Characteristics	No.of patients(%)	NIPT Positive	Prenatal diagnostic validated		PPV(%)	*P*
			**TP**	**FP**		
**Maternal age (years)**						
< 35	2605 (28.39)	16	6	9	40.00	0.142
≥ *35*	6571 (71.61)	30	13	9	59.09	
**Gestational age at NIPT (weeks)**						
9–12^+6^	7913 (86.24)	40	17	15	53.13	0.929
13–14^+6^	634 (6.90)	5	2	2	50.00	
15–19^+6^	601 (6.55)	1	0	1	0.00	
≥ 20	28 (0.31)	0	0	0	N/A	
**BMI**						
< 18.5	738 (8.04)	7	2	4	33.33	0.531
18.5–22.9	5367 (58.49)	22	10	8	55.56	
23.0–24.9	1463 (15.94)	8	5	1	83.33	
25.0–29.9	1317(14.35)	9	2	5	28.57	
≥ 30	291 (3.17)	0	0	0	N/A	
**Method of conception**						
Natural	6773 (73.81)	35	14	15	48.28	0.672
IVF	2403(26.19)	11	5	3	62.50	
**Clinical indication**						
Advanced maternal age (age ≥ 35 years)	6312 (68.79)	30	13	11	54.17	0.147
High or critical risk of serological screening	80 (0.87)	0	0	0	N/A	
Abnormal ultrasound screening	136 (1.48)	1	1	0	100.00	
Previous history	95 (1.04)	0	0	0	N/A	
Parental chromosomal aberration	24 (0.26)	2	0	0	N/A	
Voluntary demand	2326 (25.35)	13	5	7	41.67	
Others	203 (2.21)	0	0	0	N/A	

*NIPT* noninvasive prenatal testing, *TP* true positive, *FP* false positive, *PPV* positive predictive value, *IVF* in vitro fertilization, *BMI* body mass index

### Pregnancy outcome and follow-up for positive SCA NIPT

The available clinical outcomes were reviewed. Table S1 presents the ultrasound findings, karyotype test results, and pregnancy outcomes for the 37 participants who underwent invasive diagnosis after positive NIPT results for fetal SCAs.

**Fig. 1:**
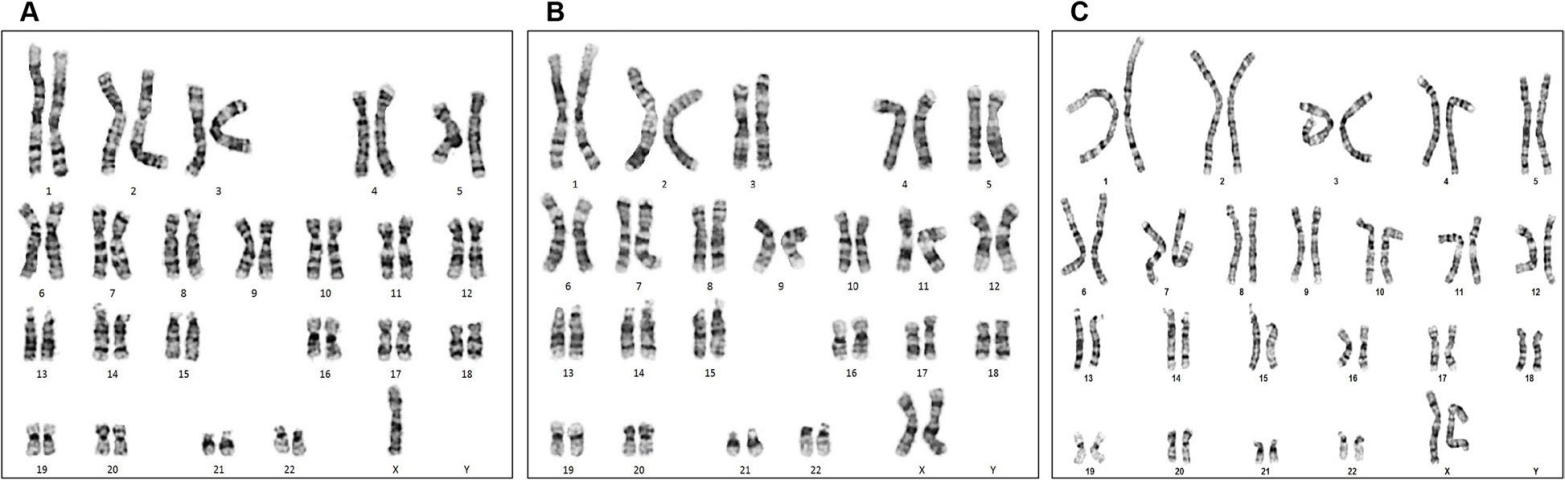
Karyotype analysis results for case 2. **A** and **B** show a karyotyping analysis of amniotic fluid, which revealed a 45,X mosaic karyotype (mos 45,X[3]/46,XX[12]). **C** Karyotype analysis of neonatal blood showed a 46,XX

**Fig. 2:**
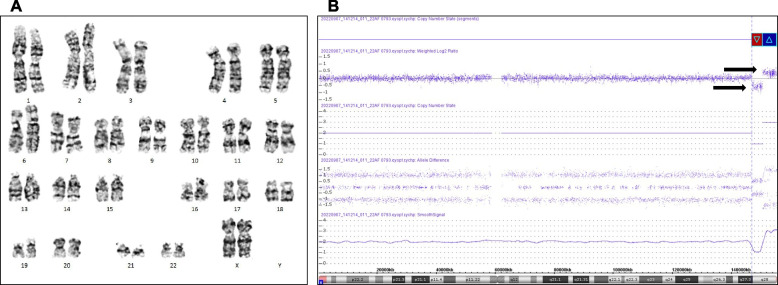
Karyotype and CMA results of case 16. **A** Karyotyping analysis of amniotic fluid, which showed a 46,XX karyotype. **B** CMA results of amniotic fluid. The arrow indicates that the X chromosome had a 3.6 Mb deletion in the Xq27.3q28 region and a 4.8 Mb duplication in the Xq28 region

Most pregnant women did not undergo maternal serum screening, and no specific abnormalities were observed on ultrasound. Among the 19 true positive cases with concordant fetal karyotype and NIPT results, fetal mosaicism was confirmed in four cases, and copy number variation was confirmed in one. Low-level mosaicism detected in this study was 20% and 15.8% for 45,X in cases 2 and 5, respectively. Postnatal karyotype on blood confirmed 46,XX in case 2 (Fig. 1). In case 16, a 3.6 Mb deletion in the Xq27.3q28 region and a 4.8 Mb duplication in the Xq28 region were identified as pathogenic variants by CMA (Fig. 2). After genetic counseling, the pregnant woman chose pregnancy termination at 21 weeks. Case 17 was identified as having a false positive NIPT result owing to maternal SCA.

## Discussion

This study evaluated the clinical performance of NIPT for SCAs. Among the 9,176 pregnancies, 46 were identified as SCA-positive through NIPT, with 37 (80.43%) undergoing invasive prenatal diagnosis. Our data indicated an overall SCA incidence of 0.207% (1/483), with specific rates of 0.033% (1/3059), 0.087% (1/1147), 0.054% (1/1835), and 0.033% (1/3059) for 45,X, 47,XXX, 47,XXY, and 47,XYY, respectively. The PPV of NIPT for SCAs in this study aligns with previous studies, which have reported PPVs of 40–55% for SCA [1, 5, 13, 15–17]. However, some studies reported higher PPVs of 70–86.7%, and a meta-analysis by Gil et al*.* demonstrated a detection rate of 90.3% for 45,X and 93.0% for other SCAs (47,XXX, 47,XXY, and 47,XYY) [12, 14, 18, 19]. Conversely, another study reported PPVs below 35% for SCAs [20–24]. Variations in SCA detection rates using NIPT among research groups have sparked controversy regarding NIPT accuracy in predicting fetal SCA, and some authors present concerns about the expanded use of NIPT in the general population [15, 26, 27]. Differences in PPVs between studies may stem from the limited number of SCA cases in each study and variations in sample size, clinical characteristics, sequencing depths, and algorithms used [25].

In this study, 18 cases showed discrepant results between NIPT and prenatal diagnosis, and these false positives can be attributed to fetal and maternal factors. Fetal factors affecting NIPT accuracy include a low fetal fraction, confined placental mosaicism, and vanishing twins [28]. Although it was excluded from this study due to criteria, we observed cases where the actual genetic status of the fetus was missing due to fetal factors. The maternal age at the time of NIPT was 38 years, with a gestational age of 12^+5^ weeks and a BMI of 25.6. However, a no-call result was reported due to the low fetal fraction (3.36%). Subsequently, the karyotype of the fetus confirmed 45,X through amniocentesis. The study of Hui et al*.* supports this discrepancy that failed results due to low fetal fraction was associated with a higher risk of aneuploidy, ranging from 2.7% to 23.3% across several sequencing platforms [29]. NIPT reflects the genetic status of the placenta because it uses cffDNA derived from apoptotic trophoblast cells in placental tissue. This can lead to false positive NIPT results by missing the actual genetic status of the fetus [30]. Another potential mechanism for false positives is the early demise of a co-twin affected by monosomy X. Maternal factors affecting NIPT accuracy include maternal SCAs or mosaicism, copy number variations, and maternal malignancy [31, 32]. Abnormal maternal chromosomes influence NIPT results because most cfDNA in maternal plasma consists of maternal DNA, except for a small amount of fetal DNA (3–10%) [33, 34]. Recent studies by Wang et al. and Lu et al. reported that 8.6% and 12.5% of SCA-positive results are attributed to maternal sex chromosome mosaicism [5, 35]. In our study, maternal peripheral blood karyotype analysis was performed in 16 of the 46 SCA-positive cases, and maternal SCA and mosaicism were identified in 5 (10.86%). The fetus in case 17 showed a normal karyotype of 46,XX in the amniocentesis, discordant with the SCA-positive NIPT result. Later, the mother was confirmed 47,XXX. Additionally, maternal SCA and mosaicism were confirmed in four out of nine pregnant women who tested positive for fetal SCA through NIPT but did not undergo invasive diagnosis (mos 45,X [34]/46,XX[66] in one case, 47,XXX in three cases). Those results might be due to the limitation of shotgun sequencing methodology for NIPT that cannot directly distinguish cffDNA from maternal backgrounds, underscoring the importance of pre-and post-test counseling [36].

Consistent with previously reported studies, PPV for 45,X was much lower than that for other SCAs in this study [1, 5, 13, 15, 16]. Several factors contributed to the poorer performance of NIPT in screening for monosomy X as opposed to trisomies. The X and Y chromosomes have 58 homologous genes, with 29 genes located at both ends of the pseudoautosomal region. Highly homologous regions may likely lead to a reduction in mapping quality. Additionally, the low guanosine-cytosine content of the X chromosome leads to highly variable amplification of the X chromosome [20, 37, 38]. The low PPV for monosomy X may also be attributed to the non-random X chromosome inactivation in placental tissue, where the paternal X chromosome may be inactivated in XX female trophoblasts [5, 37]. Additionally, age-related loss of X chromosomes in normal female white blood cells has been reported, which could affect the prediction of fetal 45,X [39].

Nondisjunction, which can occur during meiosis or in the early stages of post-junction development, is a well-known cause of SCA. Age-related aneuploidy is associated with X-chromosome centromere dysfunction resulting from premature centromere division [40]. Despite several investigations, the association between maternal age and fetal SCAs remains inconclusive, owing to conflicting findings in most studies. In previous studies comparing fetal SCA incidence through prenatal diagnosis in pregnant women of different ages, not limited to AMA women, Li et al*.* observed significant correlations between maternal age and 45,X and 47,XXY incidences. However, no correlation was observed for 47,XXX and 47,XYY [41]. In contrast, a few studies reported that 47,XXX and 47,XXY incidences significantly correlated with maternal age, whereas 45,X and 47,XYY incidences did not show significant correlations [5, 42]. However, our findings indicated that fetal SCA incidence was not significantly associated with maternal age (*P* = 0.914), except for the borderline significance of 47,XYY (*P* = 0.048). Some possible confounding factors may explain these conflicting outcomes. First, paternal age may be considered. Nondisjunction events responsible for losing a paternal sex chromosome are the most common genetic mechanisms, accounting for approximately 70–80% of 45,X monosomy cases. Moreover, over half of the 47,XXY karyotypes result from paternal errors at meiosis I, whereas the remaining cases originate from maternal meiosis I or II or postzygotic mitotic errors. In contrast, 47,XYY can exclusively arise from paternal errors, either at meiosis II (approximately 85%) or from postzygotic events [2, 43]. Second, preemptive elimination of aneuploidy by preimplantation genetic testing for aneuploidy (PGT-A) may be considered. PGT-A is widely used to detect aneuploidy in embryos to improve implantation rates after IVF, and some studies have reported that it can reduce aneuploidy and miscarriage rates [44, 45]. Interestingly, 26.19% (2,403 women) enrolled in this study were IVF pregnancies, showing a much higher proportion than that of other studies (5.9%, 2.66%, and 10.0%) [16, 17, 31]. Among these, 5.99% (144 women) had undergone PGT-A, implying that more aneuploidy might have preemptively filtered out via PGT-A before NIPT screening. This reduced number of incidences may become a confounding factor in observing an association between AMA and fetal SCAs.

According to several studies, aneuploidies are known to be more likely to occur in cases with AMA, an earlier gestational age, a history of a trisomic pregnancy, and pregnancy with a female fetus. Additionally, it was reported that pregnancy with a history of IVF-ET was more likely to have T18 and SCAs [46, 47]. Maternal obesity also increases the risk of pregnancy complications, such as spontaneous abortion and congenital anomalies, but obesity alone is not known to be a risk factor for fetal aneuploidy [48]. However, this study did not identify a correlation between maternal risk factors and fetal SCAs.

We evaluated the PPVs of all fetal SCAs using NIPT. Our data suggest that NIPT can be reliable for screening SCAs, particularly in predicting sex chromosome trisomies compared with monosomy X. No significant correlation existed between pregnancy characteristics, including maternal age and fetal SCA incidence. However, there are still some limitations. The sample size was relatively small, and the number of SCA cases was limited, making it unsuitable to determine the correlation between fetal SCAs and maternal age. Moreover, our study had a higher proportion of AMA at 71.61% compared with other studies, and the relatively early timing of sample collection for NIPT may have influenced our results. Access to clinical follow-up information was unavailable for some SCAs, preventing the confirmation of pregnancy outcomes. Placental testing for confirming confined placental mosaicism did not proceed to all patients with SCA-positive.

## Conclusion

The accuracy of these findings requires improvements; however, our study can provide an important reference for clinical genetic counseling and further management. This study is limited by a relatively small sample size, a small number of SCA cases, and challenges in placental testing for false positive cases. Therefore, further studies with larger sample sizes, considering confounding factors such as paternal age and whether or not PGT-A has been performed, are required for more accurate evaluation.

### Supplementary Information

**Additional file 1: Supplementary Table 1.** Results and outcomes for positive SCAs NIPT.

## Data Availability

The datasets generated during the current study are available from the corresponding author on a reasonable request.

## Abbreviations

45,X: Monosomy X (Turner syndrome)
47,XXX: Trisomy X (triple X syndrome)
47,XXY: Klinefelter syndrome
47,XYY: XYY syndrome (Jacob’s syndromes)
AMA: Advanced maternal age
BMI: Body mass index
cffDNA: Cell-free fetal DNA
cfDNA: Cell-free DNA
IVF: In vitro fertilization
NIPT: Noninvasive prenatal testing
PPV: Positive predictive value
SCA: Sex chromosome aneuploidy
PGT-A: Preimplantation genetic testing for aneuploidy
