# Supplementary material for: Clinical evaluation of noninvasive prenatal testing for sex chromosome aneuploidies in 9,176 Korean pregnant women: a single-center retrospective study
Source: BMC Pregnancy Childbirth. 2024 Jan 31;24:93. doi: 10.1186/s12884-024-06275-8 (PMC10829263; doi:10.1186/s12884-024-06275-8)
Supplement: Supplementary file 1 — Additional file 1: Supplementary Table 1. Results and outcomes for positive SCAs NIPT. [file 12884_2024_6275_MOESM1_ESM.docx]

**Supplementary Table 1. Results and outcomes for positive SCAs NIPT**

| **Case** | **Maternal age** | **NIPT results** | **MSS** | **NT (mm)** | **Second trimester ultrasound** | **Fetal karyotype** | **Maternal karyotype** | **Pregnancy outcome** | **Results** |
| --- | --- | --- | --- | --- | --- | --- | --- | --- | --- |
| 1 | 34 | 45,X | ND | 1.4-1.5 | normal | 46, XY | ND | Normal male | discordant |
| 2 | 35 | 45,X | ND | 1.4 | normal | Amniotic fluid: mos 45,X[3]/46,XX[12] Neonatal blood: 46,XX | ND | Normal female | concordant |
| 3 | 34 | 45,X | ND | 1.1 | normal | arr(1-22)x2,XY | ND | f/u loss | discordant |
| 4 | 39 | 45,X | ND | 1.5 | normal | 46, XY | ND | Normal male | discordant |
| 5 | 38 | 45,X | ND | 1.1 | normal | mos 45,X[3]/46,XX[16] | 46, XX | Healthy female | concordant |
| 6 | 35 | 45,X | ND | 1.2 | normal | 46, XX | 46, XX | Normal female | discordant |
| 7 | 38 | 45,X | ND | 1.6-1.7 | normal | 46, XX | ND | Normal female | discordant |
| 8 | 35 | 45,X | ND | 0.9 | normal | 46, XX | ND | Normal female | discordant |
| 9 | 29 | 45,X | ND | 1 | normal | 46, XY | ND | f/u loss | discordant |
| 10 | 31 | 45,X | ND | 1.5 | normal | 46, XX | 46, XX | Normal female | discordant |
| 11 | 38 | 45,X | ND | 1 | normal | 46, XY | 46, XX | Normal male | discordant |
| 12 | 31 | 45,X | ND | 1.5-1.6 | normal | 46, XX | ND | Normal female | discordant |
| 13 | 39 | 45,X | ND | 1.9 | normal | 46, XX | ND | Normal female | discordant |
| 14 | 28 | 45,X | ND | 0.8 | normal | 46, XX | 46, XX | Normal female | discordant |
| 15 | 33 | 45,X | ND | 1 | Placenta previa marginalis | 46, XX | ND | Normal female | discordant |
| 16 | 38 | 45,X | ND | 1.2 | NA | 46,XX arr Xq27.3q28(146,806,191_150,386,543)X1 [P] / arr Xq28(150,408,165_155,233,731)X3 [P] | arr(1-22)x2 | TOP | concordant |
| 17 | 38 | 47,XXX | ND | 0.8 | normal | 46, XX | 47,XXX | Normal female | discordant |
| 18 | 32 | 47,XXX | ND | 0.9 | NA | 47, XXX | 46, XX | TOP | concordant |
| 19 | 38 | 47,XXX | ND | 1 | NA | 47, XXX | 46, XX | f/u loss | concordant |
| 20 | 32 | 47,XXX | Low risk | 3 | normal | 47, XXX | ND | Healthy female | concordant |
| 21 | 37 | 47,XXX | ND | 1 | normal | mos 47,XXX[28]/46,XX[2] | 46, XX | Healthy female | concordant |
| 22 | 40 | 47,XXX | ND | 0.8 | normal | 47, XXX | ND | Healthy female | concordant |
| 23 | 39 | 47,XXX | ND | 1.4 | normal | 47, XXX | ND | Healthy female | concordant |
| 24 | 32 | 47,XXX | ND | 1.4 | normal | 47, XXX | ND | Healthy female | concordant |
| 25 | 40 | 47,XXX | ND | NA | Single umbilical artery (Rt side) | 47, XXX | ND | Healthy female | concordant |
| 26 | 39 | 47,XXY | ND | 0.9 | NA | mos 47,XXY[6]/46,XY[26] | 46, XX | f/u loss | concordant |
| 27 | 38 | 47,XXY | ND | 1.4 | Low-lying placenta | 47, XXY | ND | f/u loss | concordant |
| 28 | 40 | 47,XXY | ND | 1.8 | normal | 46, XY | ND | f/u loss | discordant |
| 29 | 40 | 47,XXY | ND | 1.7 | NA | 47, XXY | ND | TOP | concordant |
| 30 | 37 | 47,XXY | ND | 1.4 | NA | 47, XXY | ND | f/u loss | concordant |
| 31 | 34 | 47,XXY | ND | 1.3 | normal | 46, XY | ND | Normal male | discordant |
| 32 | 35 | 47,XXY | ND | 2.6 | normal | 47, XXY | ND | Healthy male | concordant |
| 33 | 36 | 47,XYY | NA | NA | NA | 46, XY | ND | f/u loss | discordant |
| 34 | 33 | 47,XYY | ND | 1.5 | normal | 47, XYY | ND | f/u loss | concordant |
| 35 | 32 | 47,XYY | ND | 1.5 | normal | 47, XYY | 46, XX | Healthy male | concordant |
| 36 | 33 | 47,XYY | ND | 1.4 | normal | 46, XY | ND | f/u loss | discordant |
| 37 | 30 | 47,XYY | Low risk | 2 | NA | 47, XYY | ND | f/u loss | concordant |

MSS, maternal serum screening; NT, nuchal translucency; ND, not done; NA, result not available; f/u loss, follow up loss; TOP, termination of pregnancy
